# Dynamics of Immune System Gene Expression upon Bacterial Challenge and Wounding in a Social Insect (*Bombus terrestris*)

**DOI:** 10.1371/journal.pone.0018126

**Published:** 2011-03-29

**Authors:** Silvio Erler, Mario Popp, H. Michael G. Lattorff

**Affiliations:** Institut für Biologie, Molekulare Ökologie, Martin-Luther-Universität Halle-Wittenberg, Halle (Saale), Germany; University of Poitiers, France

## Abstract

The innate immune system which helps individuals to combat pathogens comprises a set of genes representing four immune system pathways (Toll, Imd, JNK and JAK/STAT). There is a lack of immune genes in social insects (e.g. honeybees) when compared to Diptera. Potentially, this might be compensated by an advanced system of social immunity (synergistic action of several individuals). The bumble bee, *Bombus terrestris*, is a primitively eusocial species with an annual life cycle and colonies headed by a single queen. We used this key pollinator to study the temporal dynamics of immune system gene expression in response to wounding and bacterial challenge.

Antimicrobial peptides (AMP) (abaecin, defensin 1, hymenoptaecin) were strongly up-regulated by wounding and bacterial challenge, the latter showing a higher impact on the gene expression level. Sterile wounding down-regulated TEP A, an effector gene of the JAK/STAT pathway, and bacterial infection influenced genes of the Imd (relish) and JNK pathway (basket). Relish was up-regulated within the first hour after bacterial challenge, but decreased strongly afterwards. AMP expression following wounding and bacterial challenge correlates with the expression pattern of relish whereas correlated expression with dorsal was absent. Although expression of AMPs was high, continuous bacterial growth was observed throughout the experiment.

Here we demonstrate for the first time the temporal dynamics of immune system gene expression in a social insect. Wounding and bacterial challenge affected the innate immune system significantly. Induction of AMP expression due to wounding might comprise a pre-adaptation to accompanying bacterial infections. Compared with solitary species this social insect exhibits reduced immune system efficiency, as bacterial growth could not be inhibited. A negative feedback loop regulating the Imd-pathway is suggested. AMPs, the end product of the Imd-pathway, inhibited the up-regulation of the transcription factor relish, which is necessary for effector gene expression.

## Introduction

Host-parasite interactions are characterized by a permanent challenge between pathogenicity factors of the parasite and resistance mechanisms of the host. Induction, specificity and memory of the immune system seem to occur following pro- and eukaryotic infections in invertebrates and vertebrates [1], [2], [3], although the proximate mechanisms might differ substantially.

Social insects seem to be especially prone to parasite attacks, as their colonies frequently contain a high density of individuals, usually showing highly related genotypes, facilitating high transmission rates. In addition, social insects have an advanced system for the maintenance of nest homeostasis which may aid the transmission of certain parasitic species within the colony. Despite these characteristics enhancing parasite intrusion several mechanisms preventing parasites from ‘breaking into the fortress’ might exist. These range from behavioural adaptations, such as a highly advanced system of nest defence and nestmate recognition to hygienic behaviour like self- and allogrooming. Additionally, group effects might exist, also known as social immunity [4].

Individual members of the colonies exhibit the above mentioned behavioural repertoire as well as an intrinsic immune system. Insects, as well as all invertebrates, have an innate immune system comparable to that of vertebrates. Typically, insects lack an adaptive immune system, although several studies have shown that memory and specificity might also occur in invertebrate immune responses [5], [6].

The innate immune system consists of two parts, the humoral and the cellular response. In general, cellular immune system components are composed of the combined action of circulating cells in the hemolymph, reactions of phagocytosis, encapsulation and melanisation. On the other hand the humoral immune response involves the production of substances with antimicrobial activity due to the activation of one or several intracellular signalling pathways as well as the action of reactive oxygen and nitrogen species. These pathways are well conserved among animals. The Toll, Imd (immuno deficiency), JNK (jun-kinase) and the JAK/STAT (janus kinase/signal transducers and activators of transcription) pathways are all characterized by extracellular signal recognition by membrane bound receptors, which might activate a signalling cascade resulting ultimately in the transcriptional activation of effector molecules in the nucleus. Amongst these effector molecules the omnipresent antimicrobial peptides (AMP) are the best studied effector molecules of the immune system. Usually, their expression is regulated via the Toll and Imd pathway due to the action of NF-κB-like transcription factors Dif or dorsal and relish, respectively. While, some AMPs are highly conserved across taxa and do not show elevated evolutionary rates between populations, a number of studies on AMP evolution within social insects showed that elevated rates of molecular evolution are possible [7]. The AMPs abaecin, apidaecin, defensin and hymenoptaecin of the Asian honeybee *Apis cerana* show 2 to 13 different alleles at the protein level suggesting an accelerated evolutionary change [8]. In wood ants of the family *Formicidae* the AMP defensin shows an accelerated evolutionary speed with 2 amino acids showing true signs of positive selection [9].

With the availability of the genome sequence of the Western honeybee (*Apis mellifera*) it has been recognized that this species has the lowest gene counts for 12 of the 17 gene families when compared with *Drosophila melanogaster* or *Anopheles gambiae*. Only one third of the immune system genes have been found in the honeybee, a fact that might be due to the enormous repertoire of behavioural adaptations or novel, so far undetected genes [10]. Similarly, recent published ant genomes (*Camponotus floridanus* and *Harpegnathos saltator*) revealed substantial overlap in immune-related genes (ca. 65%) and a reduced repertoire of immunity-related genes when compared to *Drosophila* [11]. The basic genes of the immune system pathways have been also found in bumble bees by means of EST library sequencing [12]. Nevertheless, compared to these two dipteran species social bees live in a relatively clean and saprophyte free environment. This might be important, as it has been hypothesized that the immune system pathways and their high degree of conservation across taxa might not be explained by specificity to adapted pathogens, but rather in terms of protecting insects from saprophytes, omnipresent microorganisms that mainly act as decomposers of dead organic material [1].

Solitary insects, like the beetle *Tenebrio molitor*, show an immediate response towards bacterial infections, resulting in clearance of approximately 95% of bacteria within the first 30 minutes post infection without any signs of antimicrobial activity of the hemolymph [13]. Antimicrobial activity of the hemolymph was just detectable after most of the bacteria have been eliminated. This has been explained as long term protection as well as activity against surviving bacteria [13].

There are several major differences between solitary and social insects with respect to the potential for transmission of parasites, primarily relating to the behavioural adaptations that may prevent parasite attacks and the lack of immune genes. For this reason, we test the temporal dynamics of immune system activation in a model species, the bumble bee *Bombus terrestris*, for host-parasite interactions. *Bombus terrestris* is a primitively social insect species having annual colonies headed by single queen that is mated to a single male. Colonies typically grow to a size of approximately 100–300 workers producing sexual offspring exclusively towards the end of the season.

Using controlled infections of bumble bee workers, either sterile (wounding response) or non-sterile (response towards pathogens), we aimed to identifying temporal patterns of immune system gene activation. Monitoring the gene expression pattern in defined time intervals post infection we successfully identified the pathways involved as well as highlighting the connectivity between individual immunity genes and their corresponding pathways.

## Materials and Methods

### Bumble bees and infection

Colonies of bumble bees, *Bombus terrestris*, were obtained from Koppert Biological Systems (Kempen, Germany). Colonies were kept under standard laboratory conditions [14]. Until artificial infections workers were kept in colonies. Post infection individuals were kept in small observation cages (depth 100 mm×width 130 mm×height 150 mm) at 60% relative humidity and 30°C. Individuals were fed *ad libitum* with honey.

*E. coli* (strain YM109) was cultivated in 30 ml LB medium as over night culture at 37°C. The culture was centrifuged at 3000×g for 10 min. After washing the pellet two times in 25 ml autoclaved bee ringer [15] it was resuspended in 20–30 ml autoclaved bee ringer. For injections the solution was diluted to OD_600_ of 0.1.

The experimental design consisted of three different treatment groups: the ‘bacterial challenge’ group was injected with 3 µl *E. coli* solution, the ‘wounding’ group with 3 µl of autoclaved standard bee ringer and the ‘control’ group was handled in the same way as the others excluding any injection. After ½, 1, 2, 4, 8, 12 and 24 hours all surviving workers were killed by freezing in liquid nitrogen and stored at −80°C until further processing. Each group and time point was replicated three times. All bumble bees survived the three treatments, except for one worker at 24 hours after *E. coli* injection.

### cDNA synthesis and quantitative real-time PCR (qPCR)

RNA preparation followed the protocol from [16] with one bumble bee abdomen homogenised in 600 µl QIAzol Lysis Reagent (Qiagen, Hilden, Germany). Purity of each RNA sample was determined using the absorption ratio (260/280 nm) determined by NanoDrop 1000 (Pequlab, Erlangen, Germany). cDNA was synthesised according to the manufactures instructions starting with 2 µg RNA supplemented with 30 U M-MLV Reverse Transcriptase (Promega, Mannheim, Germany) and 0.625 µg 18-mer oligo dTs (Fermentas, St. Leon-Rot, Germany).

cDNA samples were diluted 1 ∶ 50 with DEPC-water (DNase- & RNase free water). 1 µl diluted cDNA was used together with 5 µl SensiMixPlus SYBR & Fluorescein Kit (SYBR-Green) (Bioline, Luckenwalde, Germany), 0.3 µM of each gene specific primer (Table S1) and 3.4 µl DEPC-water for the gene expression assay. In order to control for PCR efficiency and individual differences of samples a set of housekeeping genes was used (see Table S1). Primers for housekeeping genes were designed using Primer 3 (v.0.4.0, http://frodo.wi.mit.edu/primer3/) using sequences deposited in GenBank under following accession numbers AF181594 (28S rRNA), AY208282 (EF1α), AF492888 (AK) and DQ468668 (ITPR).

AMPs and pathway gene primers were designed from sequenced cDNA or adopted from [17], [18]. The cDNA for sequencing was produced as described before from one *B. terrestris* worker infected with *E. coli* strain YM109. Several immune pathway genes (basket - HM143000, cactus 2 - HM143001 / HM143002, dorsal - HM143003, hem - HM143004, Kenny - HM143005, Myd88 - HM143006, prophenoloxidase - HM142999, relish - HM143007, Tak 1 - HM143008, TEP A - HM143009, Toll 1 - HM143010, Toll 6 - HM143011, all deposited in GenBank) and antimicrobial peptide genes (abaecin – GU233780, defensin 1 – GU233781, hymenoptaecin – GU233782, all deposited in GenBank) were amplified using primers from [17], [19]. qPCR primers for AMPs and immune pathway key genes (dorsal, basket, prophenoloxidase, relish, TEP A) were derived from sequenced PCR products (Eurofins MWG GmbH, Ebersberg, Germany) by using Primer 3 (v.0.4.0) [20].

Bacterial growth of *E. coli* was detected using the bacterial housekeeping gene *fadD*, as part of the fatty acid metabolism (*Ec*MLST a multilocus sequence typing database system (MLST) for pathogenic *Escherichia coli*; http://www.shigatox.net/ecmlst/cgi-bin/scheme). Gene expression of *fadD* was normalised to the initial amount of bacteria at starting point. The qPCR protocol for all primer pairs consisted of an initial denaturation step of 10 min at 95°C followed by 35 amplification cycles (95°C, 15 sec; T_annealing_, 30 sec and an elongation step at 72°C for 30 sec) and subsequent melting curve analysis between 50°C and 98°C, reading the fluorescence at 1°C increments. Two replicates for each sample were run using Chromo4™ (Bio-Rad, Munich, Germany). Samples from the same individuals and from individuals with the same measuring point or treatment were allocated on different plates, in order to minimize a between-plate effect.

### Data analysis and statistics

Opticon Monitor 3 (Bio-Rad, Munich, Germany) software was used to compute C_t_ values after baseline subtraction and PCR efficiencies were calculated using LinRegPCR [21]. Replicate samples showing a difference in C_t_ values larger than 0.5 were re-run. Finally, all replicates showed low variance for C_t_ values.

Relative gene expression (r_E_) was calculated according to this formula:
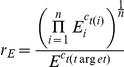

- *C_t_* = cycle threshold
- *E* = PCR efficiency
- *i* = i^th^ housekeeping gene
- *n* = number of housekeeping genes
- *r_E_* = relative expression

**Figure 1:**
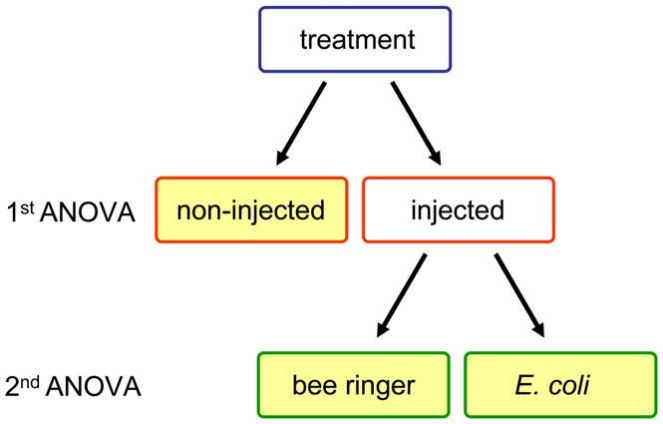
Hierarchical overview of statistical analysis for the gene expression data. Two major groups build up the analysis of treatment (non-injected vs. injected). The injected treatment is subdivided in bee ringer vs. *E. coli* as type of injection. Yellow marked groups represent the real experimental groups with gene expression data of each individual which were used for comparing relative gene expression. Two ANOVAs show the level of statistical analysis: first ANOVA, non-injected vs. injected (red boxes) and the second ANOVA, bee ringer vs. *E. coli* (green boxes).

Values for r_E_ were log-transformed in order to ensure normality and homoscedasticity of the data. Treatment, time p.t. (time point post treatment) and treatment by time interaction effects on levels of gene expression were tested using factorial ANOVA. The experimental design is hierarchical with control (non-injected) and injected individuals structuring the first level and within injected bees, discriminating between bee ringer and *E. coli* injection at the second level of the analysis (Figure 1). This scenario requires two subsequent ANOVA in order to discriminate between effects at the different hierarchical levels. In all analysis the residuals from the first ANOVA were used as values for the second ANOVA. Hence, effects of time p.t. are eliminated in this way. In case of significant interaction terms we applied a Scheffé post hoc comparison in order to determine significant differences.

Correlation analyses between groups of genes (e.g. AMPs) and single factors (e.g. bacterial growth, transcription factor activity) usually included multiple testing, which has been taken into account by using a Bonferroni adjustment of the p-values. In the case where several variables were correlated as well we used partial correlation analysis to account for this.

In order to determine which of the pathways is involved in a specific treatment, multiple regression analysis was used to determine which transcription factors that might induce the expression of effector genes. All statistical analyses were done using standard spreadsheet software and STATISTICA 8.0 (StatSoft, Tulsa, Oklahoma, USA).

## Results

### Treatment-dependent AMP expression

The response in AMP (abaecin, defensin 1 and hymenoptaecin) gene expression towards injection was considerable. Gene expression levels of all three antimicrobial peptides increased rapidly after injection. *B. terrestris* AMP expression was regulated in different intensities pending on treatment (Table 1). Pure sterile injection (wounding) leads to significant increase of expression for abaecin, defensin 1 and hymenoptaecin compared to non-injected bumble bees (p<0.001; Table 1). *E. coli* injection had a higher impact on AMP expression compared to sterile injection (bee ringer). Defensin 1 and hymenoptaecin showed stronger up-regulation for the *E. coli* treated group (defensin 1, p = 0.02; hymenoptaecin, p = 0.032). Abaecin missed significant differences by comparing both types of injection (p = 0.251), but expression is significantly effected by injection (Table 2).

**Table 1:** The effect of treatment and time post treatment on immune gene expression.

gene	effect	p-values
**abaecin**	treatment	**<0.001**
	time p.t.	0.771
	treatment×time p.t.	**0.014**
**defensin 1**	treatment	**<0.001**
	time p.t.	**<0.001**
	treatment×time p.t.	**0.004**
**hymenoptaecin**	treatment	**<0.001**
	time p.t.	**<0.001**
	treatment×time p.t.	**0.001**
**basket**	treatment	0.297
	time p.t.	0.704
	treatment×time p.t.	0.757
**dorsal**	treatment	0.802
	time p.t.	0.892
	treatment×time p.t.	0.256
**PPO**	treatment	0.949
	time p.t.	0.523
	treatment×time p.t.	0.250
**relish**	treatment	0.422
	time p.t.	**0.009**
	treatment×time p.t.	0.144
**TEP A**	treatment	**0.043**
	time p.t.	0.263
	treatment×time p.t.	**0.017**

Statistical analysis of antimicrobial peptides (abaecin, defensin 1, hymenoptaecin) and key immune pathway genes (basket, dorsal, prophenoloxidase - PPO, relish, TEP A) testing the effect of non-injected vs. injected by using factorial ANOVA (p<0.05). All significant values for treatment / injection, time p.t. and interaction are marked in bold. ‘Time p.t.’ stands for time post treatment, means analysis of changes in gene expression at a specific time point after treatment (e.g. injection).

**Table 2:** The effect of bee ringer vs. *E. coli* injection on immune gene expression.

gene	p-values
**abaecin**	0.251
**defensin 1**	**0.020**
**hymenoptaecin**	**0.032**
**basket**	**0.004**
**dorsal**	0.134
**PPO**	0.186
**relish**	0.091
**TEP A**	0.785

Statistical analysis of antimicrobial peptides (abaecin, defensin 1, hymenoptaecin) and key immune pathway genes (basket, dorsal, prophenoloxidase - PPO, relish, TEP A) testing the effect of *E.coli* vs. bee ringer using factorial ANOVA (p<0.05). Statistically significant values are marked in bold.

**Figure 2:**
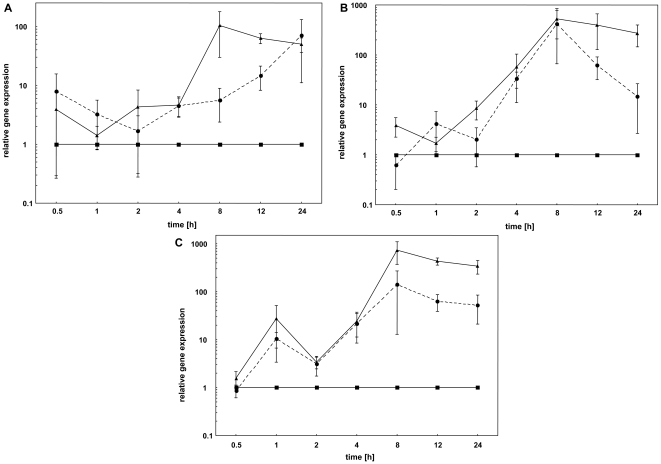
Antimicrobial peptide gene expression within 24 hours post-treatment. Gene expression pattern of abaecin (A), defensin 1 (B) and hymenoptaecin (C) in bumble bee workers within 24 hours, after injection of bee ringer (filled circles, dashed line) and *E. coli* (triangles, solid line). At each time point plots represent the median, minimum and maximum on gene expression of three workers (log-scaled, note different scales in these plots). Values for different graphs were calculated by the relation of gene expression in context to the expression level of ‘control treated’ bumble bees (squares, solid line).

Four hours post injection all AMPs deviated in their expression pattern from each other (Figure 2A–C). Abaecin and hymenoptaecin showed an increase of expression of about 10- to 50-fold between wounding and bacterial challenged samples starting at 4 hours after treatment. For defensin 1 the same pattern was observed, but starting later at 8 hours post injection.

### Immune pathway regulation

The immune system genes basket, dorsal and prophenoloxidase did not differ in their expression pattern between injected and non-injected bumble bees (Table 1). Relish (transcription factor of the Imd pathway) and TEP A (effector protein of the JAK/STAT pathway) were the only genes which showed significant differences comparing non-injected vs. injected. Gene expression of relish was significantly influenced by time p.t. (p = 0.009), but not by treatment or interaction of time p.t. and treatment. However TEP A was influenced by treatment (p = 0.043) and interaction term (p = 0.017). The Scheffé – post hoc test revealed that injection reduced TEP A expression significantly (p = 0.049). Furthermore, significant decreases for time p.t. and treatment (for injected group) were found from starting 8 hours until 24 hours post injection (Figure S1). Therefore, TEP A, a gene of the JAK/STAT pathway, was influenced by wounding and is down-regulated 8 hours post injection.

Comparison of the five target genes between *E. coli* with bee ringer injected bumble bees revealed that only basket (signalling gene in JNK pathway) was influenced significantly (Table 2). The four remaining genes showed no significant differences for treatment. Injection with *E. coli* reduced the expression of basket significantly (p = 0.004). Thus, infection with *E.coli* leads to greater down regulation of JNK pathway compared to wounding. In *Drosophila*, basket is up-regulated within the first 5 min after infection and after 1 hour no up-regulation is detectable [22].

The gene expression patterns of abaecin, defensin 1 and hymenoptaecin were correlated with dorsal and relish, the major transcription factors of the Toll and Imd pathway, respectively, which directly induce AMP expression. Only bee ringer injected bumble bees showed a significant correlation for both AMPs (defensin, p = 0.007; hymenoptaecin, p = 0.018, Bonferroni adjusted p-level) and relish. *E. coli* treatment seems to activate only defensin 1 expression (p = 0.011, Bonferroni adjusted p-level) and hymenoptaecin gave only a tendency of interaction with relish (p = 0.034). Abaecin from the bee ringer and *E. coli* group did not show any correlation neither to relish nor to dorsal (Table 3). Defensin 1 (p>0.025, Bonferroni adjusted p-level) and hymenoptaecin (p>0.025, Bonferroni adjusted p-level) indicated also no significant correlation to dorsal in both injection treatments. Significant effects of treatment, gene expression of AMPs and both transcription factors were not observed for the control bumble bee group.

**Table 3:** Correlations between transcription factors and antimicrobial peptide gene expression.

gene	treatment	abaecin		defensin 1		hymenoptaecin	
		r	p	r	p	r	p
**dorsal**	*E.coli*	−0.083	0.721	−0.105	0.651	0.005	0.982
	bee ringer	0.193	0.414	0.314	0.178	0.348	0.133
	control	0.139	0.548	0.267	0.242	0.043	0.853
**relish**	*E.coli*	0.249	0.276	0.545	**0.011**	0.464	0.034
	bee ringer	0.033	0.891	0.587	**0.007**	0.524	**0.018**
	control	−0.264	0.261	0.482	0.031	0.167	0.481

Correlations between relative gene expression of abaecin, defensin1 and hymenoptaecin, and the transcription factors dorsal and relish. Coefficient (r) and corresponding p-values are shown for all treatments. p-level was Bonferroni adjusted to account for multiple testing. p-values were Bonferroni adjusted to account for multiple testing and significant p-values are marked in bold (p<0.025).

### Immune system vs. bacterial growth

**Figure 3:**
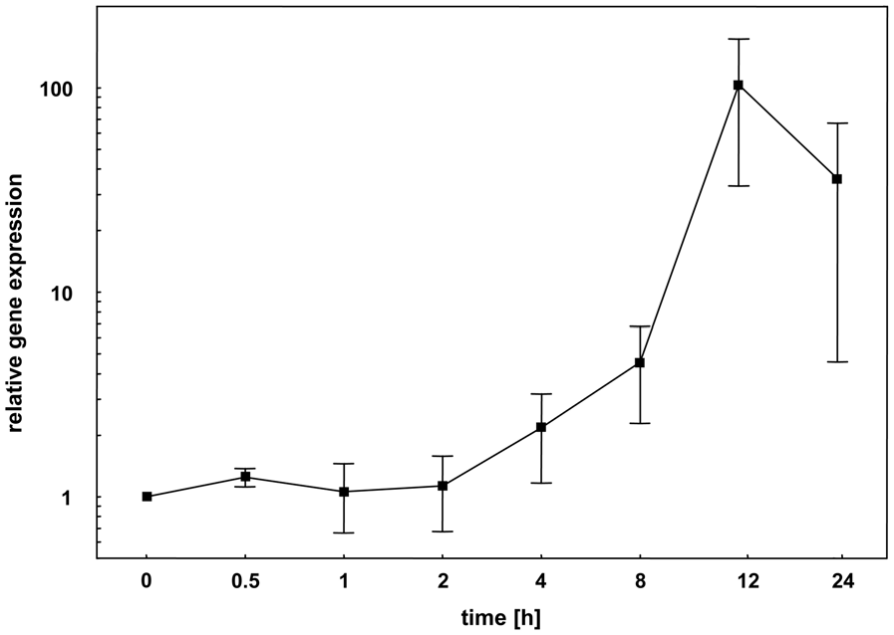
*E. coli* growth inside the host *B. terrestris* during the experiment. Temporal pattern of bacterial growth was measured, using relative gene expression of bacterial *fadD*, during the whole experiment from the starting point (0 hours) to end concentration after 24 hours post-injection. Relative bacterial growth was plotted on a logarithmic scale to show the real differences at each time point. At each time point the mean and s.e. of three individuals is shown.

**Figure 4:**
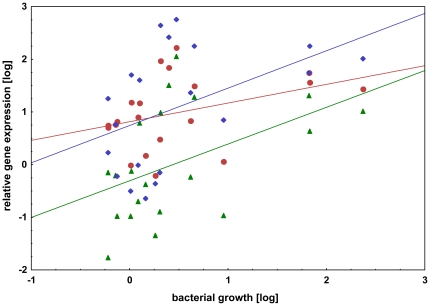
Antimicrobial peptide gene expression depending on bacterial growth. Correlation analysis between relative bacterial growth and relative gene expression of abaecin (red), defensin 1 (green) and hymenoptaecin (blue). Each data point represents one individual bumble bee with individual bacterial load and AMP expression (both were plotted with log-transformed values). Significant correlations were detected for defensin 1 (r = 0.4720, p = 0.0356) and hymenoptaecin (r = 0.4546, p = 0.0441), not abaecin (r = 0.3754, p = 0.1038).

The bacterial growth of *E. coli* was measured throughout the whole experiment (Figure 3). Within the first two hours no differences in bacterial growth between different time points were observed. However, four hours post injection bacterial growth increased strongly up to 12 hours. The amount of *E. coli* rapidly rose 10 to 100 times compare to starting concentration. Afterwards, bacterial growth decreased again. We used this to analyse a relationship between immune system gene expression and bacterial growth (Figure 4). Defensin 1 and hymenoptaecin expression correlated significantly with bacterial growth (p<0.05). For abaecin expression no significant correlation with *E. coli* growth was found, although the direction of the correlation is also positive (r = 0.375; p = 0.104).

The relative growth of *E. coli* did not significantly correlate with the relative gene expression of the five pathway genes (p>0.05, data not shown), as for antimicrobial peptide genes. However, comparison of the relative gene expression of *E. coli* injected bumble bees (after correcting for the effect of wounding) revealed two genes showing effects in relation to bacterial growth. Within the first minutes up to one hour relish had a strong expression but the level decreased as the growth rate of *E. coli* increased (Figure S2). Prophenoloxidase expression did not differ from the progression of relative bacterial growth within the first two hours. Afterwards the expression of prophenoloxidase rises well with the increasing bacterial growth (Figure S2). These relationships were not supported by significant correlations (p>0.05), but prophenoloxidase and relish expression showed a slight interaction with the infection of bumble bees by a gram-negative bacteria.

## Discussion

### Treatment-dependent AMP expression

Social insects, especially bumble bees, activate antimicrobial activity after immune challenge [19], [23], [24]. We demonstrate for the first time the treatment and time p.t. dependent expression patterns of the three antimicrobial peptides abaecin, defensin 1 and hymenoptaecin. These peptides are known to be activated by LPS (lipopolysaccharide) and PBS (phosphate buffered saline) injection. Unfortunately, many studies did not differentiate between the effect of pure injection and the effect of injection with an antigenic component or bee ringer [25]. Moreover, the different time points p.t. and duration of ‘infection’ are not comparable. Temporal pattern of immune system activation has been described for hemocyte activity in *Drosophila* [26]. Hemocytes showed temporal increases and decreases in cell numbers after wounding or injection with *E. coli*. Immune response to septic injury has also been described in variety of insects. Phenoloxidase level increased significantly after sterile and septic injury in *Drosophila melanogaster* and *Galleria mellonella* [27]. Expression of AMPs in fat body cells seems to be a combination of bacterial challenge induced pathways and cytokines activated by epithelian injury [28], [29]. We tested both components and antimicrobial peptide gene expression is up-regulated in *B. terrestris* by wounding and injection with gram-negative bacteria. However, bacterial challenge induced AMP expression to a greater extent than wounding for both defensin 1 and hymenoptaecin. Long-term expression of AMPs seems to be the best way to protect social insects against pathogen infections. Consequently, the trade-off between long-term production of antimicrobial peptides and short time activation of cytotoxic enzymes is consequently the result of natural selection balancing benefits and costs associated with protection against a broad range of parasites.

### Immune pathway regulation

Pathway activation can be described by means of immune system related gene expression. Basket, dorsal, prophenoloxidase, relish and TEP A represent five genes of the four major immune pathways of the bumble bee *B. terrestris* [12]. Concerning the low changes in expression profiles between different groups, dorsal and prophenoloxidase are not involved in wound and / or bacterial response in *B. terrestris*. Time dependent changes in the expression profile of relish did not play a key role in pathway activation after wounding. Non-significant changes or down regulation of immune pathway related genes were also observed in *Apis mellifera* [17]. For honey bees, different cluster of immune related genes were described which were down- or up-regulated after infection with different pathogens or wounding.

Treatment dependent JAK/STAT pathway activation after sterile wounding and JNK pathway activation by wounding and bacterial challenge supports our AMP expression data. Currently, the theory of wound response on injury included two pathways: JNK pathway activation in damaged tissues; and secreted unpaired cytokines activation of JAK/STAT-signalling in hemocytes and the fat body [30].

### Immune system vs. bacterial growth

A strong immune response to bacterial infection was observed in *Tenebrio molitor* [13], whereby 95% of living bacteria were killed by the immune system of *T. molitor* after injecting *Staphylococcus aureus*. The opposite case was observed for *B. terrestris* and *E. coli*, where a slight increase and decrease in bacteria number was observed during the first hours, but ultimately bacterial growth increased up to 100 times the starting concentration. Antimicrobial activity of the hemolymph in *Tribolium*, unfortunately not closely determined, can be divided in short and long time activity. Short term activity includes hemocytes and cytotoxic enzymes, on the other hand long term activity is characterised by antimicrobial peptides. Antimicrobial peptides (defensin 1, hymenoptaecin) were significantly linked to bacterial growth in *B. terrestris* over 24 hours which pleaded for pathogen specific defence regulation within host immune system. Unfortunately, we failed to connect immune system key genes with bacterial growth.

The activation of signalling molecules within the immune system pathways often involves phosphorylation or proteolytic cleavage of proteins, both post-transcriptional modifications. Changes and the transcriptional level are known for effector molecules (e.g. AMPs), but might also occur for some signalling molecules. The observed correlation of AMP expression and relish expression (Table 3) suggests a feedback loop between these components. Obviously, this feedback is activated when bees were injected, irrespective whether they received bee ringer solution or *E. coli*. Negative regulation of the Imd pathway after infection with LPS has been demonstrated to occur in *Drosophila melanogaster*. The negative feedback loop is activated after AMP expression by expression of Pirk (also known as Rudra), which acts inhibiting on the Imd-PGRP-LC complex [31], [32]. Kleino and colleagues also found a homologous sequence of the central conserved domain of Pirk for the honey bee *Apis mellifera* [31]. Hence, down-regulation of basket and relish in *B. terrestris* might be affected by negative regulation of the Imd and JNK pathway after the bacterial challenge induced AMP expression.

Additionally, two side effects were observed which fit well to the overall results. After correcting for the effect of wounding, prophenoloxidase expression followed the pattern of bacterial growth. Physiological active phenoloxidase needs to be activated by cleaving prophenoloxidase. Biochemical activity and temporal pattern of phenoloxidase is well described for *B. terrestris* [23] and it is now possible to compare expression pattern on DNA and protein level. There is evidence for a trade-off between phenoloxidase activity and antimicrobial peptides in resulting low cost control of fighting against infection.

The results of this study suggest that injection (wounding) elicits the immune system. As it is common practice to use injections of LPS in order to simulate an immune challenge, care should be taken by interpreting such results.

### Conclusions

Summarising all results, abaecin, defensin 1 and hymenoptaecin expression in *B. terrestris* is induced by sterile wounding and infection with gram-negative bacteria. Relish, the major transcription factor of the Imd pathway is highly expressed immediately after infection, but underlies negative control in context of AMP expression. Pure wounding also elicited a response by the JAK/STAT pathway. TEP A an effector gene of the JAK/STAT pathway is down-regulated by wounding.

Surprisingly, all three tested antimicrobial peptides were expressed at high levels not only after bacterial challenge but also as a response to wounding. However, bacterial challenge elicited an even stronger response in AMP expression. This suggests that the AMP expression in response to wounding is a pre-adaptation for a head start of the immune system response, as most cases of wounding under natural conditions will be non-sterile.

The complete genome sequence of the honeybee revealed a lack of immune system genes compared to other completely sequenced insect genomes. This has recently been supported by sequencing additional genomes of social insects [11]. It has been argued that the enormous behavioural repertoire of social insects, like nest defence, hygienic behaviour etc. may compensate for the lack of immune genes. We found additional evidence for differences in the ability and speed to erase invading bacterial cells between the social insect immune system compared to those from solitary insects. Haine and colleagues reported on a bacterial clearance of 95% within 30 min after exposure in the beetle *Tenebrio molitor* [13] whereas we found a constant bacterial growth in the bumble bee. There are three non-exclusive explanations for this pattern. 1) It might be due to a lack of certain immune system effector genes, 2) the activity of the innate immune system is traded-off by the highly evolved hygienic behaviour that is characteristic for certain social insects and 3) most social insects, especially bees, forage on flowers, which represent a relatively pathogen-free environment, whereas lots of other species including some of the completely sequenced insects, like *Drosophila*, live and/or forage on rotting material, which usually contains high loads of a diverse range of microorganisms.

## Supporting Information

Figure S1 **Effector protein TEP A, gene expression within 24 hours post-treatment.** Gene expression pattern of TEP A in bumble bee workers within 24 hours: after injection, bee ringer and *E. coli* were pooled together (filled circles, solid line); and non-injected (blank circles, dashed line). At each time point p.t. the median with minimum and maximum on gene expression of three workers was plotted (log-scaled). Values were calculated by the relation of gene expression in context to the expression level of ‘control treated’ bumble bees (non-injected).
(TIF)

Figure S2 **Relish and prophenoloxidase gene expression within 24 hours compared to bacterial growth.** Relative gene expression (log-scaled) of relish (empty squares, solid line) and prophenoloxidase (filled squares, dashed line). Additionally the bacterial growth (log-scaled; filled circles, solid line) during 24 hours is shown. At each time point the mean and std. error of three individuals was used.
(TIF)

Table S1 **Primer summary.** Primer used for quantitative real-time PCR with specific annealing temperatures and PCR fragment size.
(PDF)
