# Supplementary figures and images for: Dynamics of Immune System Gene Expression upon Bacterial Challenge and Wounding in a Social Insect (Bombus terrestris)
Source: PLoS One. 2011 Mar 29;6(3):e18126. doi: 10.1371/journal.pone.0018126 (PMC3066223; doi:10.1371/journal.pone.0018126)

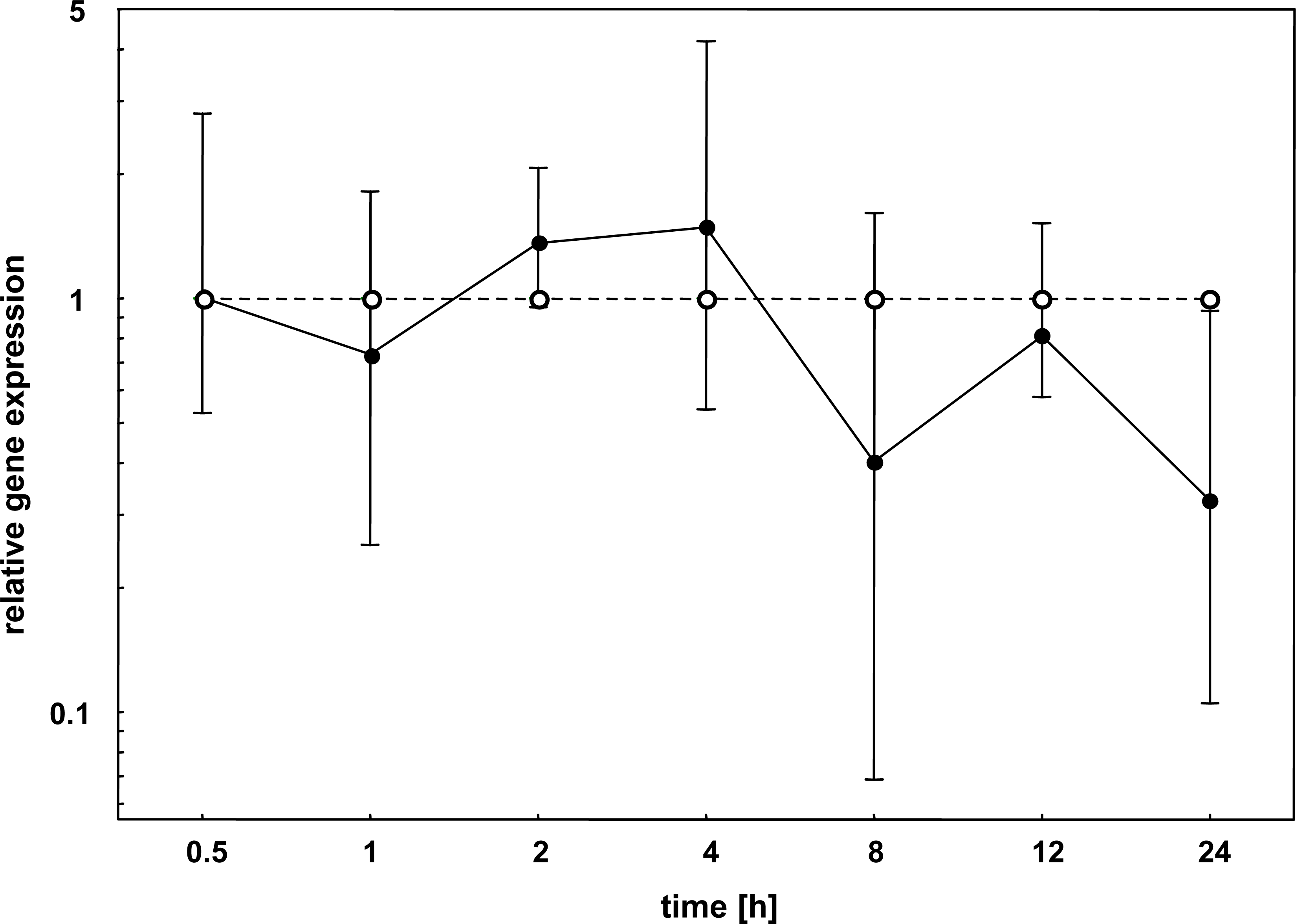

Supplement: Figure S1 — Effector protein TEP A, gene expression within 24 hours post-treatment. Gene expression pattern of TEP A in bumble bee workers within 24 hours: after injection, bee ringer and E. coli were pooled together (filled circles, solid line); and non-injected (blank circles, dashed line). At each time point p.t. the median with minimum and maximum on gene expression of three workers was plotted (log-scaled). Values were calculated by the relation of gene expression in context to the expression level of ‘control treated’ bumble bees (non-injected). (TIF) [file pone.0018126.s001.tif]

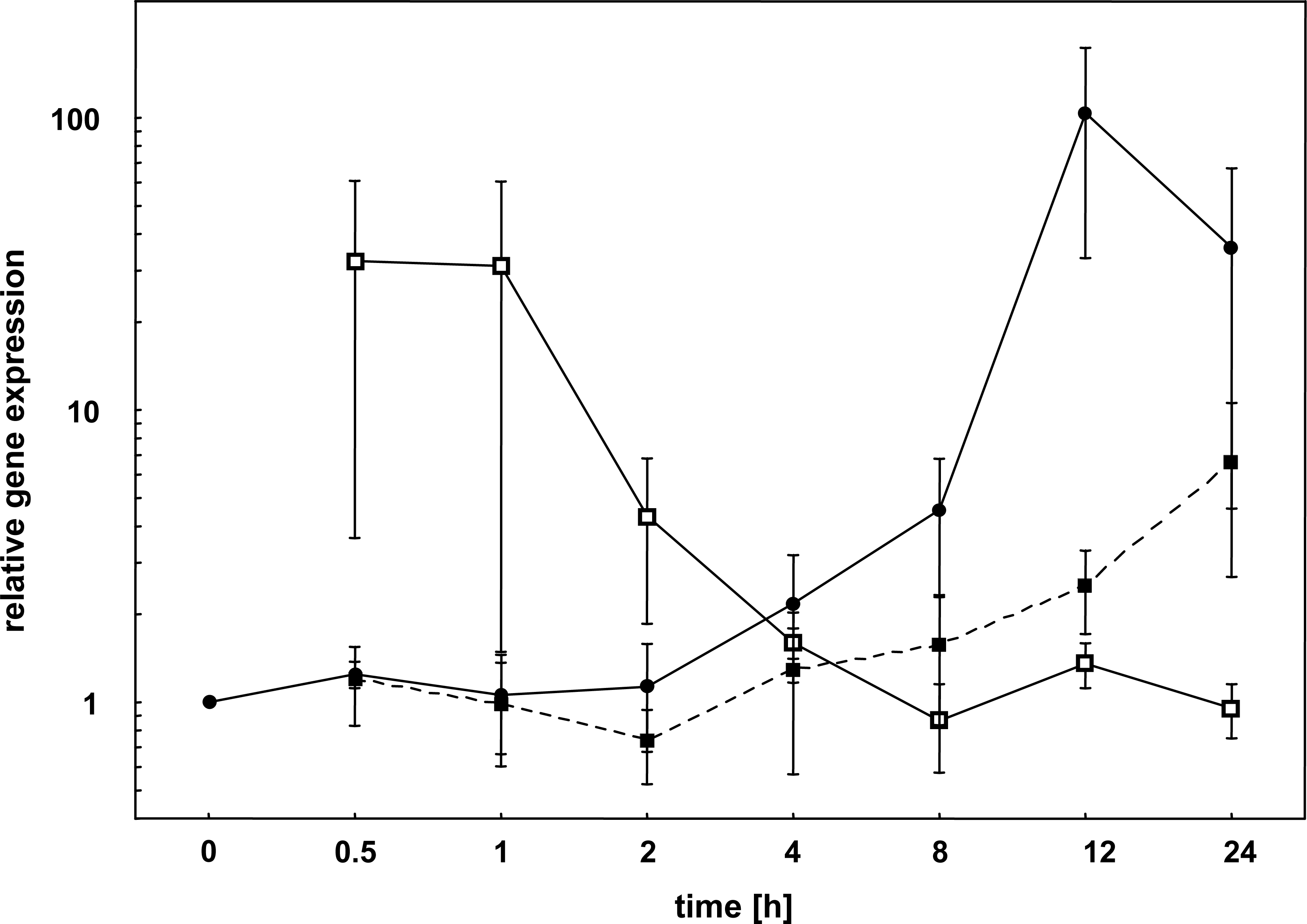

Supplement: Figure S2 — Relish and prophenoloxidase gene expression within 24 hours compared to bacterial growth. Relative gene expression (log-scaled) of relish (empty squares, solid line) and prophenoloxidase (filled squares, dashed line). Additionally the bacterial growth (log-scaled; filled circles, solid line) during 24 hours is shown. At each time point the mean and std. error of three individuals was used. (TIF) [file pone.0018126.s002.tif]
