# Supplementary material for: Dynamics of Immune System Gene Expression upon Bacterial Challenge and Wounding in a Social Insect (Bombus terrestris)
Source: PLoS One. 2011 Mar 29;6(3):e18126. doi: 10.1371/journal.pone.0018126 (PMC3066223; doi:10.1371/journal.pone.0018126)
Supplement: Table S1 — Primer summary. Primer used for quantitative real-time PCR with specific annealing temperatures and PCR fragment size. (PDF) [file pone.0018126.s003.pdf]

Table S1 Primer summary.

Primer used for quantitative real-time PCR with specific annealing temperatures and PCR fragment size.

gene:	primer:	primer sequence [5´ - 3´]:	Tm [°C]:	$T_{anneal.}$ [°C]:	size [bp]:
**abaecin**	Bt-aba-F	TGCATTTGTACCATATAATCCGCCACG	67	57.5	120
	Bt-aba-R	TGACCAGGGTTTGGTAATGGGTATGGC	70		
**defensin 1**	Bt-def-F	CGATAGACAAAGAAGAGTGA	54	57.5	155
	Bt-def-R	AGAGATCCTTGAAGTTGGT	53		
**hymenoptaecin**	Bt-hym-F	GACGACACGCTGACCCGCAA	65	57.5	252
	Bt-hym-R	CAGGACCGCGTTCAGCCTGG	67		
**basket**	Bt-bask-F	CGGGGTGGTGTGCTGTTTCCCGGC	74	53	130
	Bt-bask-R	ACGTCCAGCGGGCGCGTGTT	67		
**dorsal**	Bt-dors-F	TGCACCGGTTGCTCCACTTGCTGC	70	53	205
	Bt-dors-R	TGCGTCGGTCGCTGGTGGTATGGA	70		
**prophenoloxidase**	Bt-POO-F	CGTGGACTCGATTTCACTCCGCGTGGC	74	53	127
	Bt-POO-R	TGCGAACAGTGCCGTTTAGCGTTCCCT	71		
**relish**	Relish-F	[17]	56	53	210
	Relish-R	[17]	61		
**TEP A**	TEPA-F	[16]	56	53	190
	TEPA-R	[16]	58		
**28S rRNA**	Bt-28S-F	TCGGTCTACGGCCCGAGTGG	67	53 / 57.5	150
	Bt-28S-R	GCGGTCCAGACGCACACACA	65		
**arginine kinase**	Bt-AK-F	AAGATGCGGCCGTTCCCTGG	65	53	245
	Bt-AK-R	CAGTGGGCCAGAAGCGGCAA	65		
**EF1-alpha**	Bt-EF-F	TTCGCGGTTTCCACCGGAGA	63	53	172
	Bt-EF-R	ACGCGACCAACTGGCACTGT	63		
**Inositol 1,4,5-trisphosphate**	Bt-ITPR-F	TGCACGCAGACCAAGCGGAG	65	53 / 57.5	190
**receptor**	Bt-ITPR-R	ACGTCTTCCTTCGCGTCAAACGG	66		
E.coli fadD	fadD-F	GCTGCCGCTGTATCACATTT	58	57.5	580
**(bacterial gene)**	fadD-R	GCGCAGGAATCCTTCTTCAT	58		
